# Erythropoietin Enhances Nerve Repair in Anti-Ganglioside Antibody-Mediated Models of Immune Neuropathy

**DOI:** 10.1371/journal.pone.0027067

**Published:** 2011-10-28

**Authors:** Gang Zhang, Helmar C. Lehmann, Nataliia Bogdanova, Tong Gao, Jiangyang Zhang, Kazim A. Sheikh

**Affiliations:** 1 Department of Neurology, University of Texas Medical School at Houston, Houston, Texas, United States of America; 2 Department of Neurology, Heinrich-Heine-University Düsseldorf, Düsseldorf, Germany; 3 Department of Radiology, Johns Hopkins University, Baltimore, Maryland, United States of America; Institute Biomedical Research August Pi Sunyer (IDIBAPS) - Hospital Clinic of Barcelona, Spain

## Abstract

Guillain-Barré syndrome (GBS) is a monophasic immune neuropathic disorder in which a significant proportion of patients have incomplete recovery. The patients with incomplete recovery almost always have some degree of failure of axon regeneration and target reinnervation. Anti-ganglioside antibodies (Abs) are the most commonly recognized autoimmune markers in all forms of GBS and specific Abs are associated with the slow/poor recovery. We recently demonstrated that specific anti-ganglioside Abs inhibit axonal regeneration and nerve repair in preclinical models by activation of small GTPase RhoA and its downstream effectors. The objective of this study was to determine whether erythropoietin (EPO), a pleiotropic cytokine with neuroprotective and neurotrophic properties, enhances nerve regeneration in preclinical cell culture and animal models of autoimmune neuropathy/nerve repair generated with monoclonal and patient derived Abs. Primary neuronal cultures and a standardized sciatic crush nerve model were used to assess the efficacy of EPO in reversing inhibitory effects of anti-ganglioside Abs on nerve repair. We found that EPO completely reversed the inhibitory effects of anti-ganglioside Abs on axon regeneration in cell culture models and significantly improved nerve regeneration/repair in an animal model. Moreover, EPO-induced proregenerative effects in nerve cells are through EPO receptors and Janus kinase 2/Signal transducer and activator of transcription 5 pathway and not via early direct modulation of small GTPase RhoA. These preclinical studies indicate that EPO is a viable candidate drug to develop further for neuroprotection and enhancing nerve repair in patients with GBS.

## Introduction

Anti-ganglioside antibodies (Abs) are the most commonly recognized autoimmune markers in all forms of Guillain-Barré syndrome (GBS) [1], [2]. Association between axonal variants of GBS and specific anti-ganglioside Abs is now widely accepted [1], [3]. The full spectrum of anti-ganglioside Ab-mediated pathobiologic effects and associated mechanisms remains to be defined. Several studies suggest that GBS patients with IgG and/or IgM anti-ganglioside Abs directed against GM1 or GD1a recover more slowly and have poorer prognosis [4]–[14]. Anti-ganglioside Abs induce impairment of nerve repair is supported by our studies showing that monoclonal and patient-derived anti-ganglioside Abs inhibit axon regeneration and nerve repair after injury in an animal model [15], [16]. Further, we have established primary neuronal culture models in which anti-ganglioside Abs inhibit neurite/axon outgrowth [17]. Our cell culture studies establish that anti-ganglioside Abs induce inhibition via activation of small GTPase RhoA and its key downstream effector Rho kinase [17]. These models are not only critical to study the mechanisms underlying failure of axon regeneration in GBS cases with anti-ganglioside Abs and slow/poor recovery but they also provide an opportunity to examine therapeutic interventions to enhance axon regeneration in preclinical studies.

Erythropoietin (EPO), ∼34-kD glycoprotein, is a pleiotropic cytokine originally identified for it role in erythropoiesis [18]. It also has remarkable protective activity in preclinical models of different tissue injury. Notably, EPO has been shown to be neuroprotective in animal models of stroke, spinal cord and peripheral nerve injury, and experimental autoimmune encephalomyelitis [19]–[21]. EPO readily penetrates the blood-brain barrier (BBB) [19] and recent phase II studies showed that peripherally administered EPO is beneficial in stroke and multiple sclerosis patients [22], [23]. Some *in vitro* and *in vivo* studies suggest that EPO may promote neurite/axon regeneration in the central as well as the peripheral nervous system [24]–[27].

Since a significant proportion of cases with GBS are left with residual damage despite use of current immunomodulatory therapies, i.e., intravenous immunoglobulins and plasma exchange, the need to develop therapies to protect the neural substrate and its targets during the acute phase and enhance axonal regeneration and target reinnervation in the recovery period is increasingly realized. In view of this need, we examined the proregenerative effects of recombinant human EPO in preclinical models of inhibited axon regeneration induced with autoimmune Abs relevant to GBS. We found that EPO can significantly attenuate the anti-ganglioside Abs mediated inhibition of axon regeneration/nerve repair, and cell culture studies show that EPO induced proregenerative effect is through EPO receptor (EPOR) and sequentially activating Janus kinase 2 (JAK2)/Signal transducer and activator of transcription 5 (STAT5) pathway.

## Results

### EPO enhances neurite outgrowth of normal primary sensory and motor neurons

**Figure 1:**
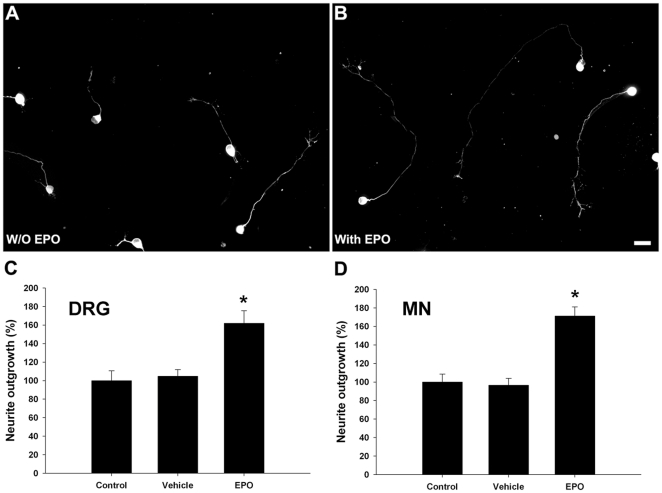
EPO enhances neurite outgrowth in control primary neurons. Primary DRG neurons without EPO treatment (A) and with EPO treatment (B). Scale bar, 20 µm. Quantified data showing that EPO treated DRG (n = 300–500 neurons) (C) or spinal motor neurons (MN) (n = 300–500 neurons) (D) have significantly longer neurites. * *p*<0.05.

To show proregenerative effects of EPO, we examined whether EPO enhances neurite outgrowth of primary dorsal root ganglion (DRG) and spinal motor neuron cultures. Motor and sensory neurons account for ∼80% and ∼50% of the total cell population in the primary spinal motor and DRG neuronal cultures, respectively. EPO (100 pM) significantly enhanced neurite outgrowth of both primary sensory (≈ 60%) (Fig. 1A–C) and motor neurons (≈ 70%) (Fig. 1D).

### EPO reverses anti-ganglioside Ab-mediated inhibition of neurite outgrowth

**Figure 2:**
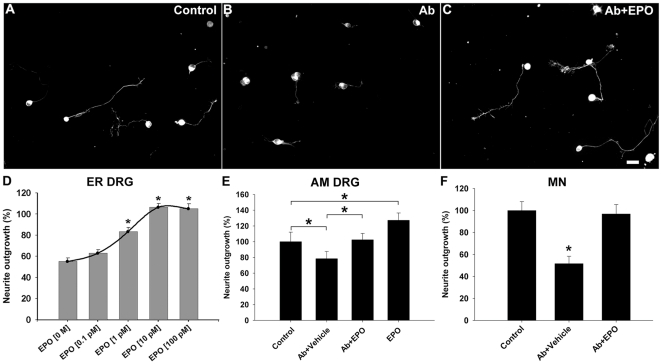
EPO reverses GD1a/GT1b–2b mAb-mediated inhibition of neurite outgrowth in primary neuronal cultures. (A) Primary DRG neurons extend long neurites under control conditions. (B) DRG neurons treated with GD1a/GT1b–2b mAb have shorter neurites. (C) EPO reverses Ab-mediated inhibition of neurite outgrowth. Scale bar, 20 µm. (D**)** EPO induces a dose-responsive reversal of Ab-mediated inhibition of neurite outgrowth in embryonic rat DRG neurons (ER DRG). Quantified data showing that EPO (100 pM) reverses Ab-mediated inhibition of neurite outgrowth in adult mouse DRG (AM DRG), * *p*<0.05 (E), and spinal motor neurons (MN), * *p*<0.01 (F).

We have previously shown that anti-ganglioside Abs inhibit neurite outgrowth in primary neuronal cultures [17], therefore, we examined whether EPO reverses this inhibitory effects of anti-ganglioside Abs. For these studies we used anti-GD1a/GT1b IgG2b (GD1a/GT1b–2b) monoclonal antibody (mAb), a prototypic antibody which has been extensively characterized for its inhibitory effects on axon/neurite outgrowth in animal and cell culture studies [15], [17]. Consistent with our previous results [17], the current studies showed that GD1a/GT1b–2b mAb significantly inhibited neurite outgrowth of both sensory (embryonic, postnatal rat and adult mouse DRG) and motor (embryonic rat) neurons in overnight cultures by 30–50% compared to control mAb-treated cultures. EPO completely reversed the GD1a/GT1b–2b mAb-mediated inhibition of neurite outgrowth in sensory and motor neuron cultures (Fig. 2). EPO dose-responsiveness was examined in primary embryonic rat DRG cultures treated with GD1a/GT1b–2b mAb, and we found dose-dependent enhancement of neurite elongation with maximal effect peaking at 10–100 pM (Fig. 2D). Since anti-ganglioside Abs and EPO induced similar effects on sensory and motor neuron cultures, remaining studies were performed on DRG cultures because of higher cell yields and easier preparation techniques.

### EPO reverses GBS sera's inhibitory effect on neurite outgrowth

**Figure 3:**
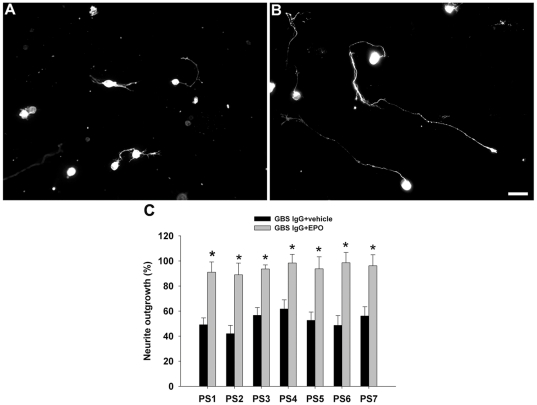
EPO reverses GBS patient IgG-mediated inhibition of neurite outgrowth in primary neuronal cultures. Photomicrographs showing that GBS IgG fractions induce inhibition of neurite outgrowth in DRG cultures (A), this is reversed by EPO (B). Scale bar, 20 µm. (C) Quantitative data showing that EPO reversed inhibition induced by seven GBS patients derived IgG fractions containing anti-ganglioside Abs. * *p*<0.05.

Our previous studies shown that IgG anti-ganglioside Abs against GD1a or GM1 present in GBS patient sera can significantly inhibit the neurite outgrowth of primary neuronal cells and the regeneration of injured axons in animals [16], [17]. In the current study, we examined whether EPO reverses the inhibitory effects of GBS patient sera on neurite outgrowth in primary neuronal cultures. GBS sera with high titers (1∶10,000 to 1∶20,000) of IgG anti-GD1a and/or GM1 Abs, collected from patients with acute motor axonal neuropathy (AMAN) during the acute phase of the disease, were used in the cell culture studies. The clinical, electrophysiological and serological features of these patients were published previously [28]. We found that IgG fractions obtained from GBS sera induced significant inhibition of neurite outgrowth in DRG neurons (Fig. 3A) compared to IgG fractions from control sera (Fig. 3B). This result is consistent with our previous findings showing that GBS sera induce inhibition of axon regeneration in an animal model [16]. Our results show that the treatment with EPO almost completely reversed neurite outgrowth inhibition induced by IgG fractions obtained from GBS sera (Fig. 3C). These studies indicate that EPO reverses not only the anti ganglioside mAb-mediated outgrowth inhibition, but also the inhibition induced with patient Abs.

### EPO-induced neurite outgrowth is mediated by JAK2/STAT5 pathway through EPOR

**Figure 4:**
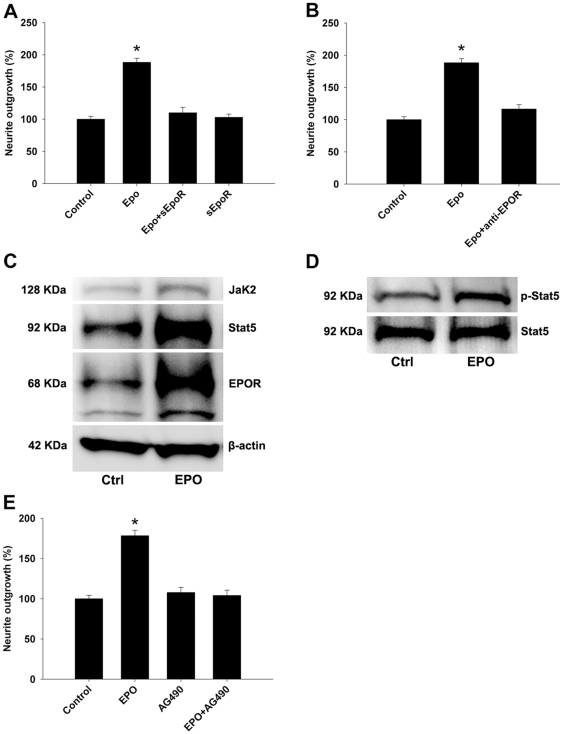
The neurotrophic effects of EPO on primary DRG neurons are mediated via EPOR. Neurotrophic effects induced by EPO are abolished by co-incubation with soluble EPOR (10 µg/ml; sEpoR) (A), and also significantly inhibited by anti-EPOR blocking Abs (5 µg/ml) (B). (C) EPO induces tyrosine-phosphorylation of several proteins in primary DRG cultures and protein migration corresponding to JAK2, STAT5, and EPOR. (D**)** EPO induces phosphorylation of STAT5 in DRG cultures. (E**)** JAK2 inhibitor (AG490; 25 µM) itself did not affect neurite outgrowth in DRG cultures, however, it did prevent enhancement of EPO-induced neurite outgrowth of DRG neurons. Control (Ctrl)  =  Vehicle {1X PBS for EPO; DMSO (0.0005%) for AG490} + medium. * *p*<0.01.

This set of studies evaluated the role of EPOR and JAK2/STAT5 pathway in EPO-mediated proregenerative effects in our cell culture models, because EPOR is expressed on motor and sensory DRG neurons [29]–[32] and JAK2/STAT5 are known to be involved in EPOR mediated intracellular signaling [33], [34]. In our initial immunocytochemical studies, we confirmed that EPOR is expressed by primary neurons in culture (data not shown). To test the role of EPOR in the enhancement of neurite outgrowth in our cell culture models, we applied soluble EPOR or anti-EPOR blocking antibody [34] in DRG cultures. Our results show that soluble EPOR competitively abolishes EPO's neuroregenerative effect (Fig. 4A). Additionally, EPO's proregenerative effects were almost completely abolished with an anti-EPOR blocking antibody in this model (Fig. 4B).

Western blotting studies were performed to assess the role of JAK2/STAT5 pathway in our cell culture model. We found that EPO induced tyrosine-phosphorylation of several proteins in primary DRG cells, some of which migrated to molecular weights corresponding to JAK2, STAT5, and EPOR (Fig. 4C). Phosphorylation of STAT5 in DRG cultures was confirmed with a specific antibody (Fig. 4D). Functional studies showed that JAK2 inhibitor, AG490, blocked EPO-mediated enhancement of neurite outgrowth of DRG neurons (Fig. 4E), and this blockage by AG490 is dose-dependent (data not shown).

### EPO does not modulate activation of small GTPases RhoA, Rac1 and Cdc42

**Figure 5:**
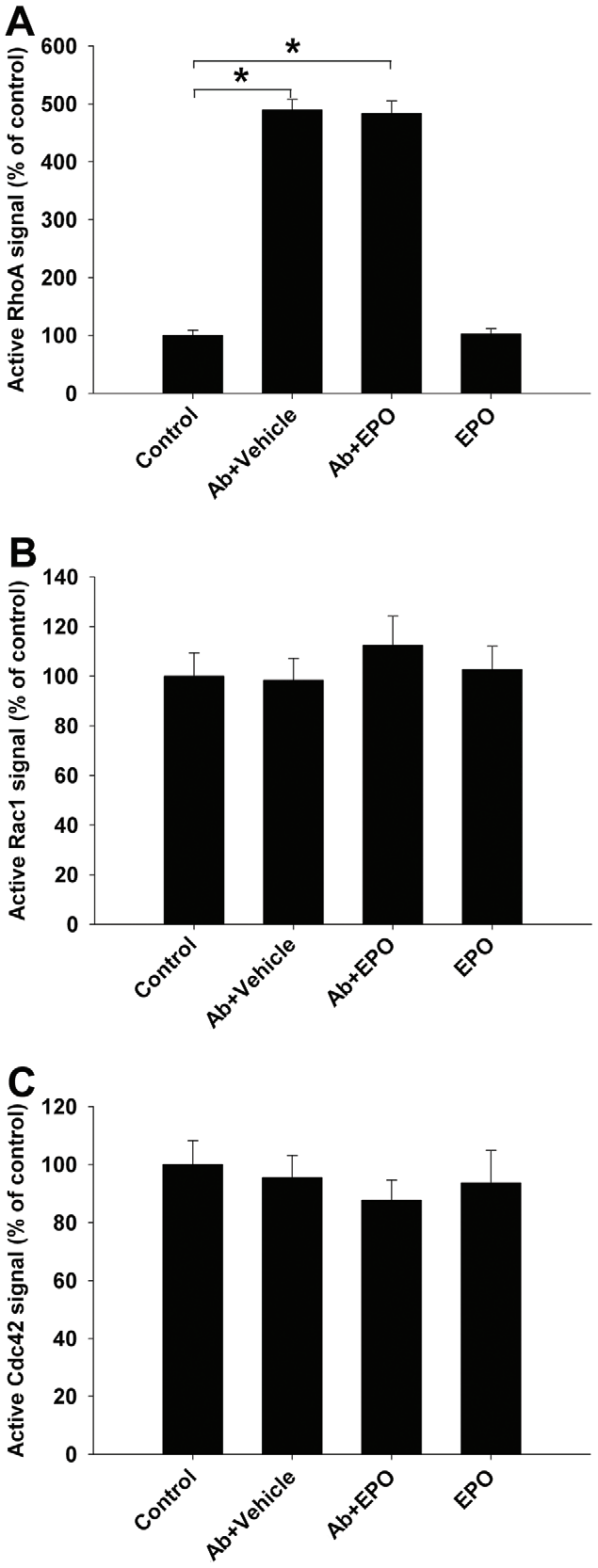
EPO does not modulate the activation of RhoA, Rac1 and Cdc42 in primary DRG cultures. (A) Anti-ganglioside Abs (GD1a/GT1b–2b; Ab) induced significant RhoA activation; co-incubation with EPO did not alter the anti-ganglioside Ab-mediated activation of RhoA. EPO did not induce RhoA activation compared to control. EPO did not modulate the activation of Rac1 (B) or Cdc42 (C) in the presence of control or anti-ganglioside Abs. * *p*<0.001.

We previously reported that specific anti-ganglioside Abs induce inhibition of neurite outgrowth through the activation of small GTPase RhoA [17]. Since EPO reverses this inhibition, we asked whether EPO modulates the activation of small GTPases (RhoA, Rac1, and Cdc42), involved in the regulation of growth cone extension [35]–[39], in our DRG cultures. We found that EPO did not alter the activation of RhoA, Rac1 and Cdc42, in the presence or absence of anti-ganglioside Abs (Fig. 5A–C).

### EPO enhances repair in an animal model of anti-ganglioside Ab-mediated inhibition of axon regeneration

We have established a standardized sciatic nerve crush model in which anti-ganglioside Abs (mAbs and GBS IgG fractions) inhibit axon regeneration [15], [16]. We next investigated whether EPO can improve axon regeneration in this animal model. These studies were limited to GD1a/GT1b–2b mAb because sufficient quantities of human sera were not available to conduct animal studies. For these studies, age and gender matched mice underwent standardized sciatic nerve crush and were evenly distributed into the following 4 treatment groups: 1) GD1a/GT1b–2b Ab + EPO; 2) GD1a/GT1b–2b Ab + vehicle; 3) Control Ab + EPO; and 4) Control Ab + vehicle. Mice were administrated GD1a/GT1b–2b mAb or control Abs on day 3 after the nerve crush by intraperitoneal route. EPO or vehicle control was administered subcutaneously to animals 5 days/week for 30 days after the nerve crush. Previously validated outcome measures included axon regeneration assessed with morphometry of sciatic and tibial nerves, and target reinnervation assessed by sciatic nerve conductions and *ex vivo* magnetic resonance imaging (MRI) of the calf muscles [15], [16], [40].

The efficacy of EPO in mice was confirmed by monitoring RBC counts in GD1a/GT1b–2b mAb+EPO (Ab+EPO) -treated and GD1a/GT1b–2b mAb+ vehicle (Ab+vehicle) groups. Our results show that Ab+EPO -treated group had significant increase in RBC counts compared to Ab+vehicle-treated animals (data not shown).

**Figure 6:**
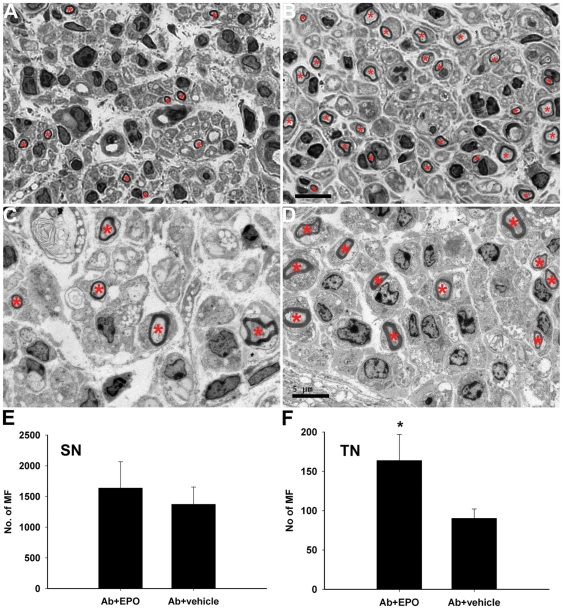
EPO enhances the number of regenerating myelinated nerve fibers (MF) in animal model. Light (A, B; scale bar, 10 µm) and electron (C, D; scale bar, 5 µm) micrographs of tibial nerve sections. Few regenerating myelinated axons (asterisks) are seen in GD1a/GT1b–2b mAb+vehicle (Ab+vehicle)-treated animals (A, C) compared to GD1a/GT1b–2b mAb+EPO (Ab+EPO)-treated animals (B, D), which have many more regenerating fibers. Quantified data show increased numbers of regenerating MF at both sciatic nerve (SN) (E) and tibial nerve (TN) (F) levels in Ab+EPO-treated group compared to Ab+vehicle-treated animals, EPO's proregenerative effect reached significance at tibial nerve level. * *p*<0.01.

Morphological studies showed that EPO treatment increased the number of myelinated regenerating axons both at sciatic and tibial nerve levels in Ab+EPO-treated group compared to Ab+vehicle-treated animals (Fig. 6). Morphometry showed that at sciatic level there were 1637±428 myelinated fibers (MFs) in Ab+EPO-treated group compared to 1373±280 MFs in Ab+vehicle-treated animals (Fig. 6E); this difference showed a trend towards significance (*p* = 0.06). At tibial level there were 164±33 MFs in Ab+EPO-treated group compared to 90±12 MFs in Ab+vehicle-treated animals (Fig. 6F); this difference was highly statistically significant (*p* = 0.001). The observation that Ab+EPO group had significant difference at tibial (distal branch) but only trend towards significance at sciatic level (proximal nerve) is consistent with the injury paradigm, i.e., more regenerative fibers close to the crush site reach sciatic level than the distal tibial level. Our previous study had shown that more profound inhibitory effect by anti-ganglioside Abs can be found at tibial nerve compared to the sciatic nerve level [15]. The results reflect either more regenerating fibers and/or faster rate of regeneration in the Ab+EPO group manifesting as more robust growth at tibial/distal level. Overall, our study indicates that EPO enhances nerve repair in this model of antibody-mediated inhibition of axon regeneration.

**Figure 7:**
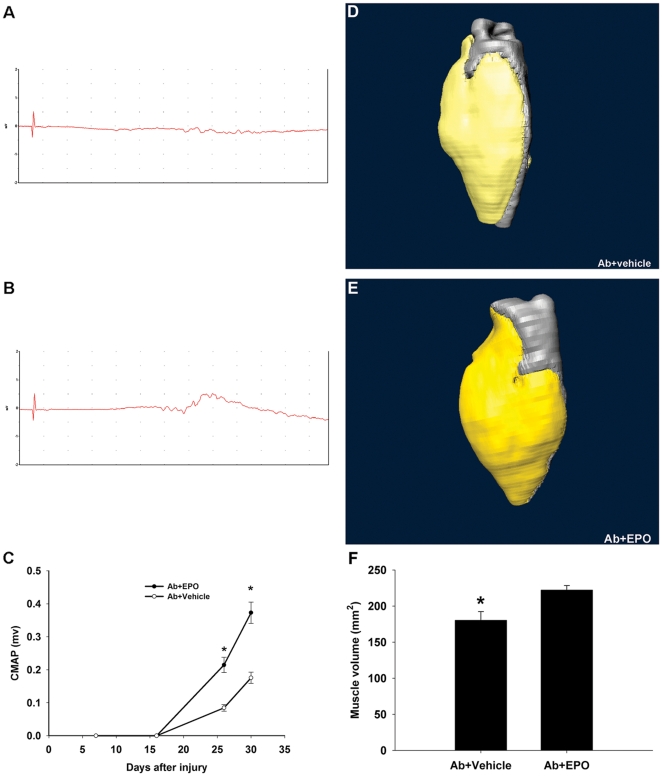
EPO enhances target/muscle reinnervation as assessed by nerve conductions and MRI volumetric measurements. Representative CMAP recordings from GD1a/GT1b–2b mAb+vehicle (Ab+vehicle)-treated animals (A) and GD1a/GT1b–2b mAb+EPO (Ab+EPO)-treated group (B). (C) Quantified data showing significantly increase CMAP amplitudes in Ab+EPO-treated group compared to Ab+vehicle-treated animals on days 26 and 30 after the nerve crush. MRI 3D reconstructions of calf muscles in Ab+vehicle-treated animals (D) and Ab+EPO-treated group (E). (F) Quantified data show significantly lower calf muscle volume in Ab+vehicle-treated animals compared to Ab+EPO-treated group. * *p*<0.05.

Target/muscle reinnervation was also assessed by sciatic nerve conductions and MRI volumetrics of calf muscle in this animal model. Electrical studies showed that significantly higher proportion of the Ab+EPO-treated animals (80%) had compound muscle action potential (CMAP) responses at day 26 after the crush compared to Ab+vehicle-treated group (44%). Quantitative data showed significant differences in CMAP amplitudes in Ab+EPO-treated group (0.22±0.02 mV at day 26; 0.37±0.03 mV at day 30) compared to Ab+vehicle-treated (0.08±0.01 mV at day 26; 0.18±0.02 mV at day 30) animals on days 26 and 30 after the crush (Fig. 7C). MRI volumetric measurements of total calf musculature at the termination of study showed significantly lower total muscle volume in Ab+vehicle-treated group (180±12 mm^3^) compared to Ab+EPO-treated animals (222±6 mm^3^) (Fig. 7F). Both measures showed that EPO significantly enhanced muscle/target reinnervation.

We also examined the effects of EPO on nerve regeneration in animals treated with control (unrelated) antibody. We found that at sciatic and tibial levels there were ∼12% more MFs in Control Ab + EPO -treated group compared to Control Ab+vehicle-treated animals; this difference did not reach statistical significance (*p* = 0.07 at sciatic level; *p* = 0.06 at tibial level).

## Discussion

Our studies show that systemic administration of EPO enhances axon regeneration in clinically relevant cell culture and animal models of nerve repair produced with experimental and patient autoantibodies directed against gangliosides. The beneficial effects were observed both on nerve regeneration and target reinnervation in an animal model as assessed by morphological and functional measures. EPO is FDA-approved and one of the earliest recombinant proteins in clinical use as a drug. These preclinical studies demonstrating EPO-mediated neurotrophism and extensive past experience with this medication make it a valuable candidate to develop further as a proregenerative therapy in GBS.

The current study focused on antibody-mediated models because of the prevalence of these immune effectors in patients with GBS. The presence of anti-GM1 and -GD1a Abs is strongly associated with axonal forms of GBS, but these Abs (particularly anti-GM1 Abs) have been reported in up to 20% cases with demyelinating form of GBS (reviewed in [41]). The relevance of these Abs to induction of demyelinating forms of GBS is not clear, however, these Abs have been associated with poor recovery in demyelinating forms of GBS [4]–[6], [9]–[13], and we have shown that GBS patient derived anti-GM1Abs inhibit axon regeneration in an animal model [16]. Further, the single most important factor relevant to prognosis in all forms of GBS (including demyelinating forms) is the extent of axonal injury [42]–[44]. Therefore, we believe that the implications of these findings are not restricted to axonal forms of GBS but that they also extend to demyelinating variants of the disease. Moreover, Mausberg et al. (submitted simultaneously along with this manuscript) show EPO's efficacy in experimental autoimmune neuritis (EAN) [42], [45]–[49], a T-cell orchestrated animal model of acute inflammatory demyelinating polyneuropathy.

The signaling studies indicate that EPO modulates the growth state of the neurons in cultures without specifically reversing the inhibitory signaling induced by anti-ganglioside Abs at the time points examined. This is supported by results showing that EPO's proregenerative effects in primary DRG cultures were mediated via the activation of EPOR and its downstream signaling cascade involving JAK2/STAT5. It has been shown that binding of EPO to EPOR promotes the phosphorylation of JAK2, the phosphorylated receptor sequentially activates several signal transduction proteins, including STAT5. The activated STAT protein then binds to the promoters of specific genes in the nucleus and initiates transcription of those genes [34], [50]–[52]. Recent studies show that activation of STAT5 is essential for the neurotrophic effects of EPO on neurite outgrowth [34], [53], which is consistent with our results. The exact signaling cascade downstream of JAK/STAT, which promotes neurite outgrowth, is not completely characterized but some studies have suggested that PI3K/Akt pathway is partially involved in EPO induced neuroprotective and neuoregenerative responses [24], [53]. We have previously shown that anti-ganglioside antibody-mediated activation of small GTPase RhoA is an early signaling event (within 30 minutes) that induces inhibition of neurite outgrowth [17]. However, EPO did not directly alter the anti-ganglioside antibody-mediated early activation of small GTPases RhoA at the time points examined (up to 30 minutes) indicating that modulation of small GTPases (RhoA, Rac1, and Cdc42) is not an early signaling event underlying proregenerative effects of EPO in DRG neuronal cultures. Since small GTPases RhoA, Rac1, and Cdc42 are considered critical and essential mediators of growth cone extension in neurons [35]–[39], it would not be surprising if these signaling molecules were indirectly affected later in EPO-treated cultures as our phenotypic studies typically assess the neurite length after overnight treatment with Abs and/or EPO. Alternatively, EPO could indirectly modulate the downstream effectors of RhoA. These signaling issues are beyond the scope of the current study.

The assertion that EPO modulates the growth state of the neuron in these preclinical studies is also supported by our animal model showing that animals treated with control Ab and EPO had increased numbers of regenerating fibers at both sciatic and tibial levels compared to the group treated with control Ab and vehicle alone; however, this relationship did not reach statistical significance due to small numbers of animals in each experimental group. Our studies with anti-ganglioside Abs show that EPO can overcome inhibition induced by these Abs in sciatic nerve injury model. These animal findings suggest that EPO has proregenerative effects that can overcome immune inhibitory signaling. However, these neurotrophic effects are not specific to immune injury and EPO could enhance nerve repair in other neuropathic conditions. This is corroborated by other studies showing that EPO is neuroprotective in metabolic and toxic models of neuropathy [25], [26], [54]. The ability of EPO to promote axon regeneration/nerve repair remains under-exploited [24] and our studies add to the existing data on proregenerative effects of EPO on neurite/axon growth in the peripheral nervous system. These data support the possibility that EPO or its non-erythropoietic forms/analogs [33], [34], [55] could be useful drugs for enhancing nerve repair in immune and non-immune neuropathic conditions.

### Clinical implications

GBS remains a major public health burden because a significant proportion of patients require mechanical ventilation and 20% have severe and permanent neurologic sequelae, including 10% who cannot walk unaided [3], [56], [57]. Patients with residual deficits and significant disability almost always have axonal injury and target denervation [43]; recovery thus requires regeneration from the site of axonal transection. Neuroprotective agents that can limit the amount of nerve injury during the disease phase and promote the nerve repair/axon regeneration during the recovery phase of GBS are extremely desirable because they can limit the permanent neurologic sequelae in patients with GBS.

In contrast to chronic neurological disorders, neuroprotective and neurotrophic strategies are more likely to yield benefit in GBS for the following reasons: a) the aberrant autoimmune responses causing nerve injury are self-limited in GBS; b) peripheral nerves have an inherent ability to regenerate and repair themselves after injury; c) there is breakdown of blood-nerve barrier in injured nerves, and neuroprotective drugs administered during the acute phase of the disease are likely to reach the injured nerve fibres; and d) neuroprotective and neurotrophic intervention(s) will be for a limited period (because of the monophasic nature of GBS) and thus less likely to lead to untoward side-effects that can potentially appear with long term use of such drugs. EPO is a viable candidate because of extensive clinical experience with its use in the treatment of hematologic/anemic disorders [58] and substantial preclinical and emerging clinical experience with its use for the treatment of neurological disease models/disorders [33]. The two potential limiting side-effect of chronic use of EPO for neuroprotection are increased risk of thromboembolism and its proproliferative effects on preexisting neoplasia [59]. These side effects are less likely to be a major issue in GBS because it is anticipated that in this condition use of EPO or other proregenerative agents would only be necessary for a limited/finite period to enhance nerve repair, and patients with preexisting cancer can be excluded from receiving this treatment. An alternate strategy would be to consider non-erythropoietic forms of EPO, which retain their neuroprotective properties but lack proproliferative effects on myeloid tissues and are projected not to induce thromboembolism [55].

In summary, now there is evidence that specific anti-ganglioside Abs can injure intact nerve fibers [60]–[62] and also impair axon regeneration via activation of specific signaling pathways that inhibit regeneration [15]–[17]. The current therapies clearly modulate the humoral and cellular immunity. Taken into account the proregenerative effects of EPO in the cell culture and animal models, underlines our concept, that addition of neurotrophic therapies such as EPO, has the potential to enhance nerve repair in patients with GBS.

## Materials and Methods

### Ethics Statement

All animals were handled according to protocols that were approved by the Animal Welfare Committee at The University of Texas Health Science Center at Houston (Protocol number: HSC-AWC-08-071 and HSC-AWC-08-062) and that are in accordance with Federal guidelines.

### Anti-ganglioside monoclonal antibody

GD1a/GT1b–2b, a prototypic anti-ganglioside mAb well characterized in cell culture and animal models [15], [17], [62]–[64], was used in these studies. We have demonstrated that this mAb severely inhibit axon regeneration in the sciatic nerve crush model [15] and neurite outgrowth in primary neuronal cultures [17]. The generation, specificity, production, and purification of this mAb were reported previously [63]. The hollow fiber supernatant containing GD1a/GT1b–2b (∼3 mg/ml) was used in all animal studies; purified GD1a/GT1b-2b was used in all cell culture studies.

### GBS and control sera

Seven GBS sera with high titers of IgG anti-GD1a and/or GM1 Abs, collected from patients with AMAN during the acute phase of the disease, and five control sera collected from normal healthy controls without ganglioside reactivity were used to study their effects on primary rat DRG neurons. The IgG fractions from sera were prepared with Protein G Sepharose column (GE healthcare, Piscataway, NJ, USA) according to manufacturer's instructions. After purification the anti-ganglioside reactivities of IgG fractions were reconfirmed with ELISA, as described [17].The studies with GBS sera were restricted to neuronal cultures because large quantities of sera are necessary to conduct animal studies.

### Primary DRG neurons culture

Our previous study showed that anti-ganglioside Abs can inhibit the neurite out-growth of neurons in the dissociated neuronal cell cultures from embryonic (E15), postnatal (P5–8) rat DRG, and adult mouse DRG [17]. EPO's effect in reversing the anti-ganglioside Abs mediated inhibition was therefore tested in those primary DRG neuronal cultures, which were prepared as described [17], [65]. For the rat DRG cultures (embryonic and postnatal DRGs), the dissociated neurons were plated on Poly-D-Lysine (50 µg/ml; Sigma-Aldrich, St. Louis, MO)-coated glass coverslips at low densities (5,000 cells/well) in 24-well plates (Becton Dickinson, Franklin Lakes, NJ), and maintained in neurobasal medium (Invitrogen, Carlsbad, CA) containing 0.25% (v/v) heat-inactivated Fetal Bovine Serum (FBS) (Hyclone, Logan, UT), 2 mM L-glutamine, 2% B27-serumfree supplement (Invitrogen), and 50 ng/ml of nerve growth factor (Sigma-Aldrich). The DRG cultures from adult mouse were maintained in F12 culture medium (Invitrogen) containing 10% FBS plus 1% penicillin/streptomycin (Invitrogen), and the neurons were placed onto Poly-D-Lysine coated coverslips at 10,000 cells per well.

### Primary motor neuron enriched cultures

Spinal motor neurons cultures were prepared as described [17], [66] with minor modifications. E15 Sprague-Dawley rat spinal cord were harvested and cleared of pia-arachnoid, grey matter was harvested and cut into small pieces; this chopped tissue was digested with 0.05% trypsin and DNAse (3∶1), digestion was terminated (L15+40%FBS) and followed by gentle trituration with Pasteur pipette and differential centrifugation. The non-neuronal glial cells only account for less than 20% of the total cell population in this motor neuron enriched culture. The dissociated cells were then plated on collagen coated glass cover slips into 24 well plates (5,000 cells/well) overnight. The growth medium contains neurobasal medium supplemented with various additives (which were all from Sigma-Aldrich unless otherwise stated) including 2% B27 supplement (Invitrogen), 2.5 mg/ml albumin, 2.5 µg/ml catalase, 0.01 mg/ml transferrin, 15 µg/ml galactose, 6.3 ng/ml progesterone, 16 µg/ml putrescine, 4 ng/ml selenium, and 1X penicillin/streptomycin. These mixed dissociated cultures were stained with SMI-32 Abs (Sternberger Monoclonals, Lutherville, MD) and used for studies described below.

### Cell culture treatment with anti-ganglioside Abs and EPO

For all experiments GD1a/GT1b–2b (50–100 µg/ml) or control IgG2b mAb (Abcam, Cambridge, MA), GBS or control IgG fractions (100–500 µg/ml) were added 30 min after plating the cells. Cell cultures were treated with either recombinant human EPO (0.1–100 pM; EMD Chemicals, Gibbstown, NJ) or vehicle (1X PBS; equal volume). After overnight incubation, cells were fixed with 4% paraformaldehyde, stained with mAbs against beta-III tubulin (1∶5000; Promega, Madison, WI) or SMI-32 Abs, and developed with specific secondary Abs conjugated to fluorophores (1∶200; Molecular Probes, Eugene, OR). Images were acquired from randomly selected fields (n = 8–12) at low magnifications with a fluorescent upright microscope (Zeiss) by using 20x lens; the longest neurite of each neuron was measured and 80–120 neurons were included per condition. Neurite outgrowth was quantified with IMAGE J, a public domain image-processing program (http://rsb.info.nih.gov/ij/) or Axiovision (Zeiss), as described [17]. Each experimental condition was done in duplicate wells and repeated at least 3 times with neurons from a different set of animals. Neurite length for each condition was normalized to the control and expressed as relative neurite outgrowth. Controls included isotype-matched sham Abs, as described [17].

### Determination of EPOR and JAK2 activation in EPO-mediated neurite elongation

These studies were performed to examine whether EPOR and its downstream effectors are involved in overcoming anti-gnaglioside Ab-mediated inhibition of neurite outgrowth. DRG neuron cultures were performed and neurite outgrowth was assessed, as described above. Anti-EPOR blocking antibody (5 µg/ml; Santa Cruz Biotechnology, Santa Cruz, CA) [34], or AG490 (JAK2 inhibitor, 1–25 µM; EMD Chemicals) were added to the cell cultures 1 hour prior to treatment with EPO (100 pM for 24 h). A subset of EPO treated cultures were co-incubated with soluble EPOR (10 µg/ml; R&D Systems, Minneapolis, MN) to show that exogenous EPOR can compete with endogenous EPOR. For western blotting studies DRG cultures were treated with or without 100 pM EPO for 10 min. Cells were harvested, sonicated, and centrifuged. Protein quantification was performed on supernatants, protein (30 µg of total protein/lane) electrophoresed, and immunoblotting was performed with anti-phosphotyrosine (1∶2,000; Millipore, Temecula, CA) and anti-phospho-STAT5 (1∶500; Millipore), as described [34], [67]. Preliminary tests were conducted to determine and optimize the concentration of different reagents, including anti-EPOR antibody and soluble EPOR (data not shown).

### Determination of RhoA, Rac1 and Cdc42 activation after EPO treatment

These studies were performed to examine whether EPO modulates the activity of small GTPases RhoA, Rac1, and Cdc42 in DRG neuron cultures. DRG cultures were serum starved, EPO (10–1000 pM) was added to the cultures in the presence of control or anti-ganglioside mAbs (10 µg/ml) and control or patient derived IgG fractions (100–300 µg/ml) for various intervals (0–30 min). Cells were then lysed and activity for GTP-bound activated fraction of these GTPases (RhoA, Rac1 and Cdc42) was determined by ELISA [17].

### Sciatic nerve crush model

8 to 12-week-old wild-type (C57BL/6) were used in this study for sciatic nerve crush model, as described [15]. All animals underwent surgery, i.e., left sciatic nerve was crushed 35 mm above the middle toe for 30 s with a fine forceps on day 0, as described [15]. Age and gender matched animals were evenly distributed into following 4 treatment groups: 1) GD1a/GT1b–2b Ab + EPO; 2) GD1a/GT1b–2b Ab + vehicle; 3) Control Ab + EPO; and 4) Control Ab + vehicle. Mice were administrated a single dose of GD1a/GT1b–2b mAb (n = 18; 750ul of hollow fiber supernatant containing ∼2 mg of Ab) or control Abs (n = 18; 2 mg of purified mouse IgG) on day 3 after the nerve crush by intraperitoneal route. EPO (42 µg/kg) or vehicle control was administered subcutaneously to animals 5 days/week for 30 days after the nerve crush. The electrophysiology studies were done on days 16, 26, and 30 after the nerve crush. Studies were terminated on day 30 after the crush and nerve tissues and sera were harvested for morphological and serological analysis from all animals. Serological studies for GD1a and GT1b ganglioside binding were done by ELISA, as described [15].

### Morphometry

All animals were perfused and sciatic and tibial nerves were harvested and post-fixed in 4% paraformaldehyde overnight before processing. Sciatic nerve segments (n = 36; ∼10 mm distal to the crush site and tibial nerve segments (n = 36; ∼20 mm distal to the crush site) were embedded in Epon. One µm cross sections were stained with toluidine blue, as described [15], [68]. For quantification, all myelinated axons in a single whole cross section of the nerve were counted at light level (40X) by using a motorized stage and stereotactic imaging software (Axiovision; Zeiss, Thornwood, NY). Selected nerves (n = 6) were also examined by electron microscopy.

### Electrophysiology

Sciatic nerve conduction studies were performed on all animals. CMAP amplitudes were recorded in the hindpaw on day 16, 26, and 30 after nerve crush, as described [15].

### *Ex-vivo* Magnetic resonance imaging

A subset of animals were perfused (n = 6), and post-fixed in 4% paraformaldehyde for 24 hours at 4°C. *Ex vivo* T2-weighted magnetic resonance images of the calf region (between knee and ankle) were acquired on a vertical 11.7T NMR spectrometer (Bruker Biospec, Billerica, MA, USA) and processed, as described [40]. From 3D images of the leg, volumetric measurements of all muscle groups were obtained. We have previously validated this MRI measure and it reflects denervation and reinnervation associated changes in calf muscle and fiber size within the 4 week time period (also used in this study) in sciatic nerve injury models [15], [16], [40].

### Statistical analysis

All numerical results are presented as mean + SEM. Differences between groups were determined using ANOVA with corrections for multiple comparisons or student's *t* test, *p* values <0.05 were considered statistically significant.
